# Characterization of 3,3′-iminodipropionitrile (IDPN) damaged utricle transcriptome in the adult mouse utricle

**DOI:** 10.3389/fnmol.2024.1487364

**Published:** 2024-12-23

**Authors:** Mengyao Tian, Jingyuan Huang, Hairong Xiao, Pei Jiang, Xiangyu Ma, Yanqin Lin, Xujun Tang, Yintao Wang, Mingchen Dai, Wei Tong, Zixuan Ye, Xia Sheng, Renjie Chai, Shasha Zhang

**Affiliations:** ^1^State Key Laboratory of Digital Medical Engineering, Department of Otolaryngology Head and Neck Surgery, Zhongda Hospital, School of Life Sciences and Technology, Advanced Institute for Life and Health, Jiangsu Province High-Tech Key Laboratory for Bio-Medical Research, Southeast University, Nanjing, China; ^2^Southeast University, Shenzhen Research Institute, Shenzhen, China; ^3^School of Public Health, Tongji Medical College, Huazhong University of Science and Technology, Wuhan, China; ^4^Department of Environmental Health, School of Environmental Science and Engineering, Hainan University, Haikou, China; ^5^Co-Innovation Center of Neuroregeneration, Nantong University, Nantong, China; ^6^Department of Otolaryngology Head and Neck Surgery, Sichuan Provincial People’s Hospital, University of Electronic Science and Technology of China, Chengdu, China; ^7^Institute for Stem Cell and Regeneration, Chinese Academy of Science, Beijing, China; ^8^Beijing Key Laboratory of Neural Regeneration and Repair, Capital Medical University, Beijing, China

**Keywords:** utricle, IDPN, RNA-seq, neomycin, hair cell damage

## Abstract

Utricle is an important vestibular sensory organ for maintaining balance. 3,3′-iminodipropionitrile (IDPN), a prototype nitrile toxin, has been reported to be neurotoxic and vestibulotoxic, and can be used to establish an *in vivo* damage model of vestibular dysfunction. However, the mechanism of utricular HCs damage caused by IDPN is unclear. Here, we first studied mice balance behavior and HCs damage in IDPN utricle damage model, and found that IDPN injection *in vivo* can cause vestibular dysfunction and HCs damage, which is more pronounced than neomycin damage model. Then we used RNA-seq to characterize the transcriptome of IDPN damaged utricle in detail to identify genes and pathways that play roles in this process. We found 1,165 upregulated genes and 1,043 downregulated genes in IDPN damaged utricles, and identified that NF-κB pathway and TNF pathway may play important roles in IDPN damage model. Our study provides details of transcriptome of IDPN utricle damage model for further study of vestibular dysfunction.

## Introduction

Utricle is one of the important vestibular organs detecting linear acceleration through its sensory hair cells (HCs) in the upper layer of utricle (Lundberg et al., 2015; Wu et al., 2017). There are two main types of utricular HCs in mammalian utricle, type I HCs and type II HCs, which are different in location distribution, morphology, synaptic innervation pattern and gene expression (Burns et al., 2015; Eatock et al., 1998; Eatock and Songer, 2011). Type I HCs are predominantly found in the striolar (S) region, while Type II HCs are mainly seen in the extrastriolar (ES) region (Wang et al., 2015; Hoffman et al., 2018). Type I HCs have a distinctive long-necked, flask-shaped cell body and the base of their cell body is surrounded by and connected with calyx-like afferent nerve terminals. Conversely, type II HCs have various shapes but lack the long-necks like that of type I HCs, and are connected with general button-like afferent nerve terminals (Maroto et al., 2021; Eatock et al., 1998). Meanwhile, in adult mice, type II HCs preferentially express the calcium-binding protein calretinol (Dechesne et al., 1991; Desai et al., 2005). Damage to utricular HCs can cause vestibular dysfunction, and behavioral testing is a common method for mouse vestibular evaluation, including open field, swimming test and footprint test (Sedo-Cabezon et al., 2014; Khan et al., 2004; Boadas-Vaello et al., 2017; Wilkerson et al., 2018; Tamura et al., 2012).

Similar to cochlear HCs, utricular HCs are easily damaged by noise exposure, viral or bacterial infections, aging and ototoxic drugs (Smith et al., 2016), which leads to vestibular dysfunction (Golub et al., 2012). Clinical treatment of some diseases by ototoxic drugs such as aminoglycosides and cisplatin cause damage to utricular HCs, which leads to impaired vestibular function (Xie et al., 2011; Ishiyama et al., 2007; Callejo et al., 2017). Neomycin, one of aminoglycoside drugs, are commonly used to construct ototoxic drug damage model of utricular HCs in many research (Yoshida et al., 2015; Xu et al., 2012; Nagato et al., 2018). Neomycin can cause HCs loss, degeneration and cell–cell connection destruction (Kim et al., 2005; Monzack et al., 2015; Harris et al., 2003; Burns et al., 2012). It has been reported that neomycin may damage HCs by inhibiting K^+^ currents in type I vestibular HCs (Mann et al., 2013). Besides, HCs damage caused by neomycin has been relatively well studied, it is closely related with apoptosis which is related to Caspase pathway, JNK pathway, and NF-κB pathway (Matsui et al., 2002; Cunningham et al., 2002; Sugahara et al., 2006; Zhao et al., 2022; Kil et al., 1997; Han et al., 2018). The upstream caspase-8/9 and downstream caspase-3 are activated in utricular HCs exposed to neomycin, while only the inhibition of caspase-9 significantly protected HCs from damage by preventing the activation of downstream caspase-3 (Cunningham et al., 2002).

Nitriles, widely used in industry, can cause neurologic, hepatic, gastrointestinal, renal, and cardiovascular disorders in human and animals (Tanii, 2017). 3,3′-iminodipropionitrile (IDPN), one of nitrile compounds, has been shown to be neurotoxic and vestibulotoxic and thus cause behavioral abnormalities similar to that of the ECC (excitation, circling and chorea) syndrome (Tariq et al., 2007). IDNP can cause utricular HCs damage and can be used for an *in vivo* model of vestibular dysfunction (Zeng et al., 2020; Llorens et al., 1994; Martins-Lopes et al., 2019). According to the different exposure dose and time, IDPN can cause the degeneration of the vestibular sensory HCs and the loss of vestibular HCs through different ways such as necrosis, apoptosis and extrusion (Soler-Martin et al., 2007; Greguske et al., 2019; Seoane et al., 2001b). In the early stage of subchronic IDPN, calyx junction dismantling and reversible synaptic uncoupling occur in the vestibular sensory epithelium, followed by HCs extrusion and detachment, which eventually leads to a slowly progressive loss of vestibular function (Greguske et al., 2019; Llorens and Rodriguez-Farre, 1997; Maroto et al., 2023; Seoane et al., 2001a). Acute IDPN causes only a slight loss of neurofilaments at the afferent terminals of the vestibular calyx surrounding type I HCs, and dose-dependent degeneration of HCs, while using highest IDPN intensity, the HCs are mainly necrotic, and eventually lead to rapid loss of vestibular function (Seoane et al., 2003; Schlecker et al., 2011). The sensitivity of different vestibular organs to IDPN is different, and the sensitivity was ranked as crista, utricle, saccule from high to low, and the sensitivity of the central region of the receptors in the epithelium was higher than that in the peripheral region. Type I HCs were found to be more sensitive to IDPN than type II HCs, and the loss of HCs precedes the degeneration of nerve terminals in the acute phase of IDPN toxicity (Llorens and Dememes, 1994). Boadas-Vaello et al. (2017) discovered that although there were no variations in the vestibular toxicity of IDPN between the sexes and no variations in the systemic toxicity among 129S1 and Swiss mice, there were notable strain-dependent changes in this regard. However, IDPN at present is mostly used as a damage drug to establish utricle damage model for studying the relationship of vestibular damage, and the balance behavior dysfunction (Schlecker et al., 2011a; Sedo-Cabezon et al., 2015; Greguske et al., 2019; Llorens and Rodriguez-Farre, 1997; Llorens and Dememes, 1994a). The mechanisms and systemic characterization of transcriptome of utricle damaged caused by IDPN have not been studied.

In this study, we first systemically studied toxic effect of IDPN to utricular function and HCs damage, and found that after IDPN treatment, mice exhibited a vestibular dysregulation phenotype and utricular HCs loss, which is more pronounced than another commonly used neomycin utricular damage model. We further performed RNA-seq analysis on the IDPN-damaged utricle to identify the genes and pathways involved in regulating HCs damage after IDPN-treatment to elucidate the possible mechanism of IDPN damage. These data sets can systematically explain the detailed regulatory mechanism of utricular HCs after ototoxic drug injury, and provide a theoretical basis for drug induced vestibular disease.

## Materials and methods

### Animal

Experiments were conducted using FVB mice of both sexes. The National Institutes of Health’s criteria for the care and use of lab animals were followed in all of our animal research, and we also followed the procedures permitted by Southeast University’s animal care and use committee.

### IDPN and neomycin damage model

The IDPN damage model was constructed by intraperitoneally injecting with IDPN solution (6 g/kg body weight, 0.9%w/v NaCl was injected as control) into P30 mice for a single time, and the follow-up experiment was conducted 7 days later. The neomycin damage model was constructed by subcutaneously injecting neomycin (150 mg/kg body weight, 0.9%w/v NaCl was injected as control) into P7 mice for 7 consecutive days, and 14 days after neomycin treatment, the mice were used for follow-up experiments.

### Swimming test

Mice were placed in a 250 mm × 150 mm cage with 37°C warm water. The behavior was recorded by the camera, and the score was 0 ~ 3 according to the swimming behavior of the mice as previously reported (Hardisty-Hughes et al., 2010): normal balanced swimming (score = 0), rolling to one side and hover excessively (score = 1), stay afloat (score = 2), rolling underwater (score = 3). Total swimming scores in each group were quantified and compared using GraphPad Prism 8 software.

### Open field test

A mouse open field test chamber with a size of 600 mm (Length) × 600 mm (Width) × 300 mm (Height) was used, and a digital camera was set up 2 meters above the open field for covering whole field of vision inside of the open field. After Haixin soft Visutrack high-end animal behavior analysis software setting up, keep environment quiet, put the mice into the center of the open field test box, and then observe and record the movement trajectory of the mice for 10 min.

### Gait test

A few days before the experiment, the mice were trained so that they could walk from one side of the channel to the other at a relatively constant speed, without obvious pauses or standing. At the beginning of the experiment, red and blue printing mud were used to cover the back and front paws of mice, respectively. White A3 paper was laid on the ground, and a baffle of 100 cm (Length) × 20 cm (Width) was placed on both sides, so that mice could pass through the white paper at a relatively uniform speed. The walking footprints of mice were recorded while the mice walk through the paper.

### Immunofluorescence staining

The utricle tissue was dissected from the inner ear of experimental mice. Firstly, the utricle was fixed with fixative (4% Paraformaldehyde) for 1 h at room temperature, and washed for 3 times with 0.1% phosphate buffered saline-Triton (PBST) for 5 min each time. Then samples were blocked with blocking solution (0.02% sodium azide, 0.5% Triton X100, 5% donkey serum and 1% bovine serum albumin were mixed in pH 7.4 PBS) for 1 h at 4°C, and. Incubated with anti-Myo7a (Proteus Bioscience, #25–6,790) primary antibody 1:1,000 diluted in PBT-1 (1% bovine serum albumin, 0.1% Triton X100, 2.5% donkey serum and 0.02% sodium azide in pH 7.4 PBS) at 4°C overnight. After that, Donkey anti-rabbit Alexa Fluor 555 fluorescence-conjugated secondary antibody (Invitrogen, #A-31572) 1:400 diluted in PBT-2 (1% bovine serum albumin and 0.1% Triton X100 were mixed in pH 7.4 PBS) was used to bind with primary antibody at room temperature for 1 h. Finally, after washing for 3 times with 0.1% PBST for 5 min each time, samples were mounted with DAKO Fluorescence Mounting Medium (DAKO, #S3023). The images were taken with Zeiss LSM 700 confocal microscope. ImageJ (NIH) and Photoshop CS4 (Adobe System) were used to analyze images.

### RNA extraction and Real-time quantitative PCR

The utricle tissues were dissected and put into a 1.5 mL RNase-free EP tube with 1 mL Trizol (Thermo Fisher Scientific, #15596026) for full grinding. The tissue was ground and then centrifuged for 10 min at 14,000 rpm and 4°C. A vigorous mix was performed after adding 200 μL of chloroform to 900 μL of supernatant. The mixture was placed on ice for 5 min, and centrifuged at 14,000 rpm for 10 min at 4 ° C. Then 400 μL of the upper layer solution containing RNA was carefully removed into a new 1.5 mL RNase-EP tube, and the same volume of isopropyl alcohol was added and mixed. The samples were then kept on ice for 10 min, centrifuge at 14,000 rpm at 4°C for 10 min, and 1 mL 75% (v/v) ethanol was added and mixed after the supernatant was discarded. After centrifugation at 4°C for 5 min at 14,000 rpm, the samples were air-dried for 10 min and then 12 μL DEPC water was added to dissolve the RNA samples. The quality (OD260/280 > 1.8) and concentration of the extracted RNA (389 μg/μl in 20 μL DEPC water from 46 utricles) were detected by Ultramicro spectrophotometer (Thermo Fisher Scientific, Nanodrop 2000), and the extracted mRNA was reverse transcribed into cDNA by using reverse transcription kit (Vazyme, R312-01). Real-Time quantitative PCR (RT-QPCR) reagent SYBR Green (Vazyme, Q712-02) was used for RT-QPCR according to the manufactory’s instructions on a Bio-Rad C1000 Touch thermal cycler to quantify the gene expression levels. cDNAs from Control and IDPN group were diluted to the same concentration. The QPCR reaction system was as follows: 0.4 μL primer mixture, 5 μL 2× SYBR Green, 1 μL cDNA, and ddH_2_O up to 10 μL. QPCR conditions were an initial denaturing step of 30 s at 95°C followed by 40 cycles of 10 s denaturation at 95°C, 30 s annealing at 60°C, and 20 s extension at 72°C. Sequences of the QPCR primers are listed in Supplementary Table S1. The gene expression level was analyzed by ΔΔCT method as follows: after obtaining the gene expression CT values, these data was normalized to the *β*-Actin CT value (∆CT) in the same samples to eliminate the sample differences. Next, the ∆CT values were normalized to the control group data (∆∆CT) to compare the group differences. Finally, the 2^−ΔΔCT^ value was calculated to quantify the fold difference between the control group and IDPN group.

### mRNA-sequencing and data analysis

At 7 days after a single intraperitoneal injection of 6 g/kg body weight IDPN in P30 mice, the utricles were dissected and the saline treated group was used as the control group. The tissue frozen with liquid nitrogen was used for further mRNA extraction and deep mRNA sequencing by BGI platform. The sequencing data was filtered with SOAP nuke, and afterwards clean reads were obtained and stored in FASTQ format. The reference genome was GCF_000001635.27_GRCm39. Bowtie2 (v2.2.5) was applied to align the clean reads to the gene set, in which known and novel, coding and noncoding transcripts were included. Expression level of gene was calculated by RSEM (v1.2.8), according to the gene expression difference in different samples. Differential expression analysis was performed using the DESeq2 (v1.4.5) with Q value <0.05. And then GO^1^ and KEGG enrichment analysis^2^ were performed by using Phyper hypergeometric examination.^3^ A value of Q < 0.05 was considered statistically significant. The subsequent analysis and data mining were performed on Dr. Tom Multi-omics Data mining system.^4^

### Statistical analysis

At least three separate experiments were conducted for each condition. The data was analyzed by using the GraphPad Prism 8 software and represented by mean ± standard error (SEM). A two-tailed, unpaired student T-test was used to determine statistical significance. Values with *p* < 0.05 were considered statistically significant.

## Results

### IDPN treatment causes more severe HCs damage compare to neomycin

**Figure 1:**
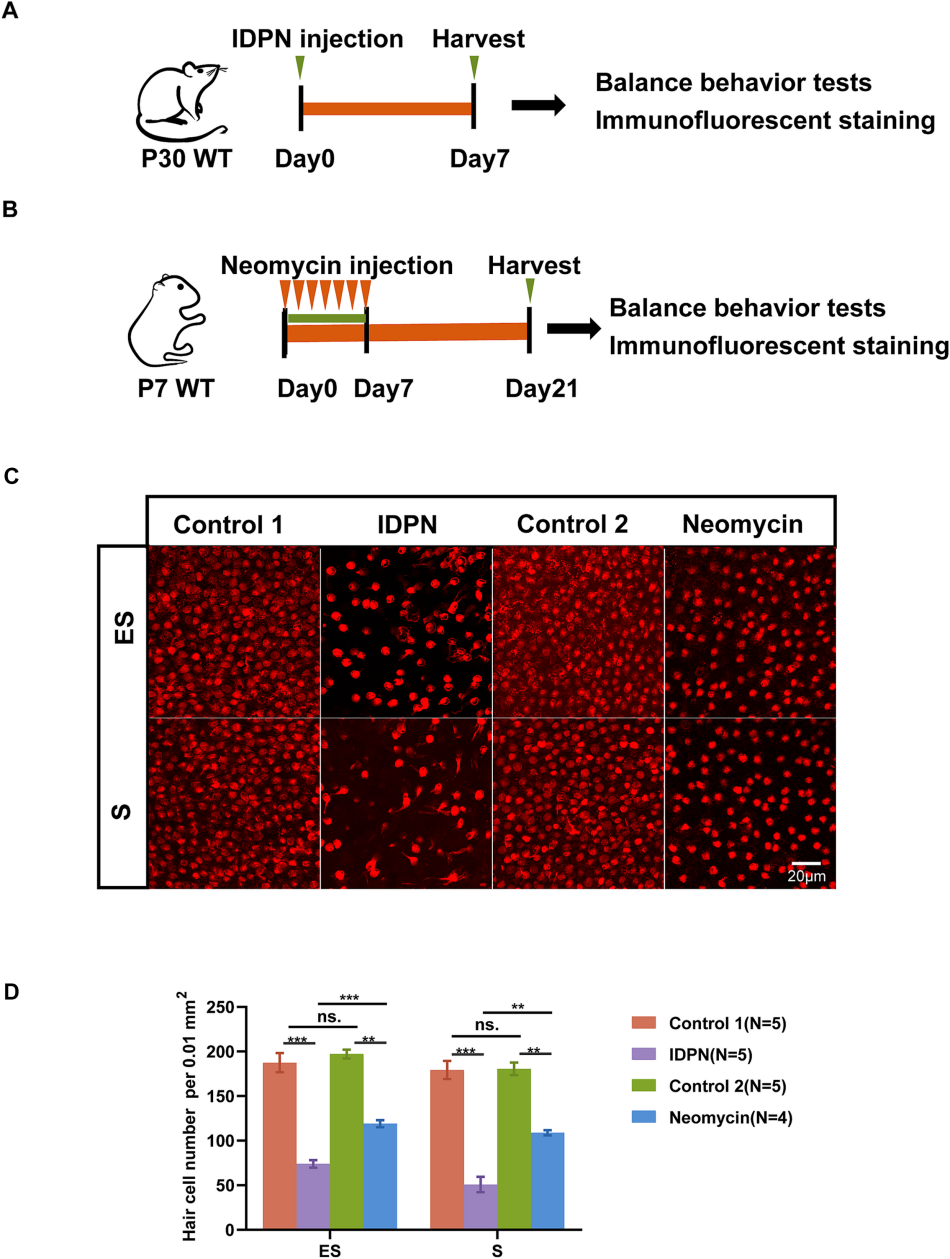
IDPN treatment causes more severe HCs damage than neomycin. **(A,B)** Schematic diagram of the *in vivo* IDPN damage model **(A)** and *in vivo* neomycin damage model **(B). (C,D)** Images **(C)** and statistical analysis of HCs number **(D)** of Striola (S) and Exstriola (ES) region of the utricle treated by neomycin and IDPN. Myo7a stained in red is used as HCs marker. Scale bar is 20 μm in **(C)** *p < 0.05, **p < 0.01, ***p < 0.001, ns, no significant difference. The number of mice (N) is shown in parentheses.

Based on previous studies, we established the utricle IDPN damage model (Figure 1A). A single injection of IDPN can cause acute injury to the utricle, leading to loss of sensory HCs. Thus, we used the commonly used dose of 6 g/kg bodyweight, administered intraperitoneally to P30 mice (Zeng et al., 2020; Martins-Lopes et al., 2019a). On the 7th day after injection, utricle was dissected, and HCs immunostaining were performed to observe the damage of utricular HCs. Furthermore, significant loss of HCs in two regions of the utricle (the S and ES regions) were observed in IDPN damage model (Figure 1C). We also established commonly used neomycin damage model by continuously injecting 150 mg/kg neomycin subcutaneously from P7 to P14. After 14 days of injection, the utricle of mice was dissected (Figure 1B). We found that although there are also HCs loss in neomycin damage model, a greater number of HCs were lost in the IDPN injury group (Figures 1C,D). Therefore, IDPN treatment causes more severe HCs damage compare to neomycin.

### IDPN treatment causes more severe balance behavioral disorders in mice

**Figure 2:**
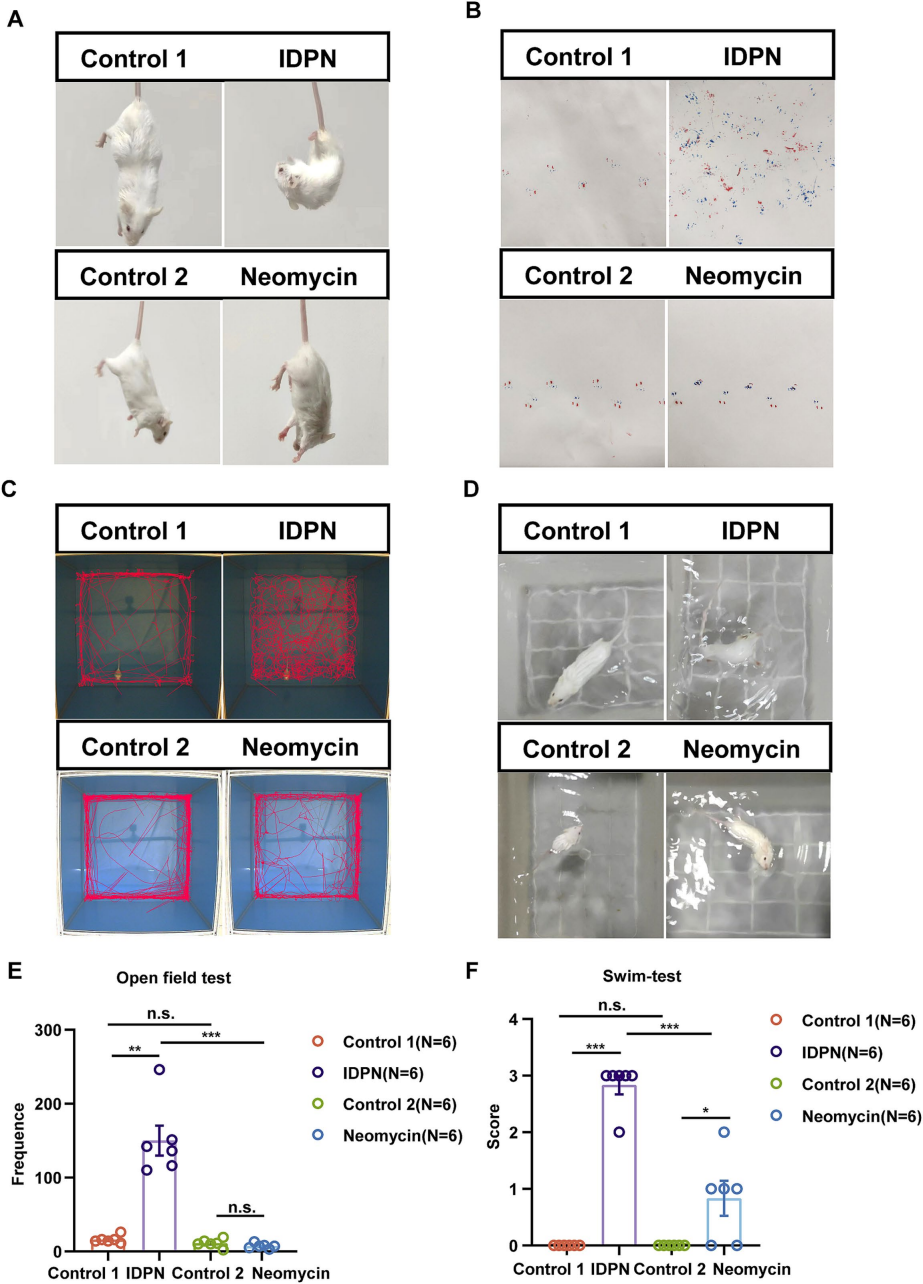
Balance behavior tests for IDPN and neomycin treatment groups. **(A)** Trunk curling tests of IDPN and neomycin treatment groups. IDPN group showed significant Trunk curling behavior. **(B)** Gait test for IDPN and neomycin treatment groups. Red, back paws of mice. Blue, front paws of mice. **(C,E)** Open field test of IDPN and neomycin treatment groups. The frequency of mice went to the center of the open field were evaluated in **(E)**. **(D,F)** Swimming test of IDPN and neomycin treatment groups. Swimming test scores were evaluated in **(F)**. *p < 0.05, **p < 0.01, ***p < 0.001. The number of mice (N) is shown in parentheses in **(E,F)**.

In order to further explore the damage effects of the IDPN damage model on vestibular function of mice, we tested the general behavior of mice by using swimming test, open field test and gait test to analyze the damage degree of vestibular function. We first observed that the mice showed trunk curling and spontaneous head shaking at day 7 after IDPN treatment, while the mice in the neomycin injury model did not show obvious behavior (Figure 2A). We then painted the front and rear paws of mouse with ink, and the mice were trained to pass through a 30 cm long white paper smoothly and leave footprints. We observed that the mice treated with IDPN could not walk straight and showed messy footprints compared to the control group, while the mice treated with neomycin showed straight footprints, but the stress on the left and right feet was slightly uneven (Figure 2B). We then performed the open field test and the movement track of mice within 10 min showed that the mice treated with IDPN had an obvious circle track, and the frequency they went to the center of the open field was significantly increased, while the neomycin model had no obvious difference from the control group (Figures 2C,E). We further used swimming test to score the vestibular function of mice. After IDPN treatment, the score was much morehigher, and most of them were rolling under water, while the mice treated with neomycin had only one slight higher swing score (Figures 2D,F). Thus, according to the results of balance behavioral experiments, IDPN treatment cause more severe vestibular dysfunction, which was consistent with the above HCs damage results.

### RNA-seq and analysis for IDPN treatment

**Figure 3:**
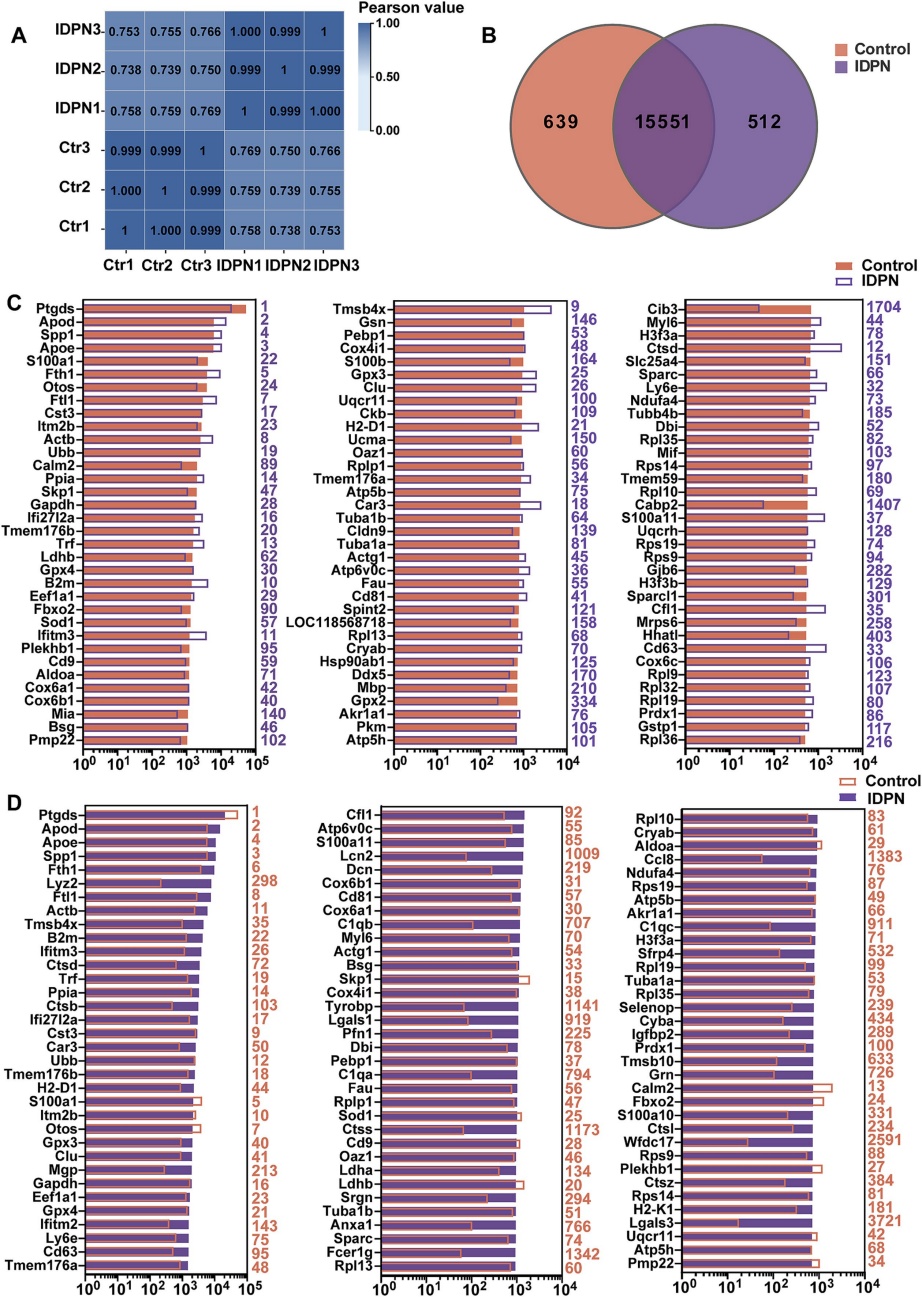
Analysis of RNA-seq data of IDPN treated and untreated utricle. **(A)** Correlation analysis of all replicated data of Control and IDPN groups. **(B)** Venn diagram showing the number of genes expressed in IDPN and Control. **(C)** The top 100 genes with high expression level in Control group in descending order. The purple number on the right side of each panel represents the same gene ranking in the IDPN group. **(D)** The top 100 genes highly expressed in IDPN group in descending order. The red numbers on the right side of each panel represent the same gene ranking in Control group.

According to the above results, treatment with IDPN seems to have more extensive and severe effects on the mouse vestibule. To investigate the detail reasons for this phenomenon and the specific mechanism of IDPN damage model, RNA-seq was performed to analyze gene expression profile of utricles after IDPN treatment. With principal component 1, the control group and IDPN group were well separated, and three replicates of each groups showed high reproducibility (Figure 3A). We measured the expression of each gene by FPKM value. After removing FPKM values below 0.1, 639 genes and 512 genes were specifically examined in the control and IDPN group, respectively, while 15,551 genes are expressed in both groups (Figure 3B).

To characterize the genes with high expression levels in control and IDPN groups, we analyzed the most abundantly expressed genes in both groups including the expression levels and abundance rankings. Figure 3C display the expression levels of the most abundant 100 genes in the control groups as shown in the red bar. We also compare the expression levels (purple bars) and abundance rankings (purple numbers) of the same genes in the IDPN groups. The expression levels of the top 100 most abundant genes in the IDPN group (purple bars) were compared with the expression levels (red bars) of the same genes in the control group and the abundance ranking (red numbers). Figure 3D shows the results of this comparison. Both figures reveal that most of the expression genes were enriched in both groups. Among the highly expressed genes, Cib3 and Cabp2 (IDPN rank >500) were uniquely expressed in the control group, and Lcn2, C1qa, C1qb, C1qc, Tyrobp, Lgals1, Lgals3, Ctss, Anxa1, Fcer1g, Ccl8, Sfrp4, Cyba, Tmsb10, Grn and Wfdc17 (Ctr rank >500) were only highly expressed in IDPN. Among these genes, Lcn2, C1qa, C1qb and C1qc are involved in inflammatory biological processes (Ghosh et al., 2020; Chen et al., 2021b; So et al., 2023), Tyrobp, Fcer1g, Wfdc17 and Lgals1, Lgals3 are involved in immune biological processes (Haure-Mirande et al., 2022; Yang et al., 2023; Xu et al., 2023; Veglia et al., 2021; Shariq et al., 2024), Tmsb10 are involved in biological processes of cell proliferation and migration (Zhang et al., 2017b), Lcn2, Anxa1, Cyba and Sfrp4 are also involved in biological processes related to inner ear (Han et al., 2012; Sena et al., 2022; Huyghe et al., 2015).

### Differentially expressed genes (DEGs) after IDPN treatment

**Figure 4:**
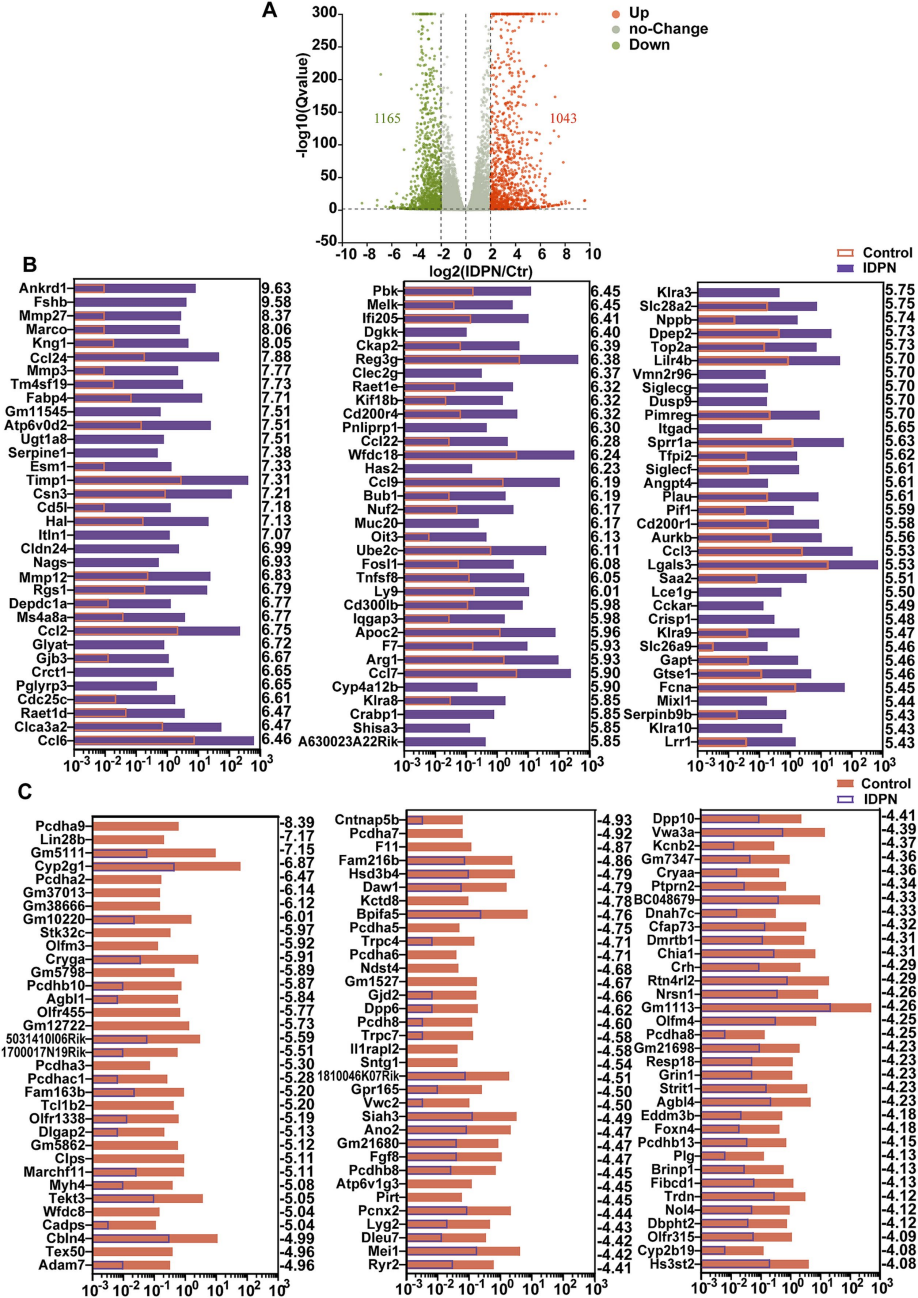
Differentially expressed genes (DEGs) in IDPN treated and untreated utricle. **(A)** Volcano map of transcripts differentially expressed in Control and IDPN groups. log2 (Fold change) > 2, Q-value < 0.05. Orange dots represent up-regulated DEGs in IDPN, green dots represent down-regulated DEGs in IDPN, and gray dots represent genes with no significant expression difference between Control and IDPN groups. **(B)** The top 100 up-regulated DEGs in IDPN group in descending order. The numbers on the right side of each panel represent the log2 (FoldChange). **(C)** The top 100 down-regulated highly DEGs in Control group in descending order. The numbers on the right side of each panel represent the log2 (FoldChange).

To identify differentially expressed genes between control and IDPN groups, we compared their gene expression levels and screened for DEGs [log2(fold change) > 2, Q value<0.05]. These genes are depicted in volcano map, including expression up-regulated 1,165 genes and down-regulated 1,043 genes in IDPN group (Figure 4A). Figure 4B shows the top 100 up-regulated genes in IDPN group, and these upregulated genes have been extensively studied in immune inflammatory response, neuronal development, and cell death, including ankyrin repeat domain 1 (Ankrd1) (Shen et al., 2015), Tissue inhibitors of metalloproteinases1 (Timp1) (Hu et al., 2012; Eisner et al., 2017), C-C motif chemokine ligand 2(Ccl2) (Chen et al., 2022; Sautter et al., 2006), C-C motif chemokine ligand 6 (Ccl6) (Coelho et al., 2007; Nakagawa et al., 2019), FOS-like antigen 1 (Fosl1) (Xu et al., 2018a; Vaz et al., 2012; Bakiri et al., 2014), etc. Figure 4C shows the top 100 down-regulated genes in IDPN group, and some of which, such as Siah3 (Abd Elghani et al., 2022), olfactomedin4 (Olfm4) (Gong et al., 2021), and fibroblast growth factor 8 (Fgf8) (Ratzan et al., 2020) have been confirmed to play an essential role in negative regulation of cell death and synapse formation. This supports our view that IDPN causes severe damage to the utricle by affecting aspects of inner ear development, neuronal development, cell death, as well as immune inflammatory responses. However, quite a few DEGs have not been previously reported in utricle and need to be further investigated in the future.

### GO and KEGG analysis of DEGs after IDPN treatment

**Figure 5:**
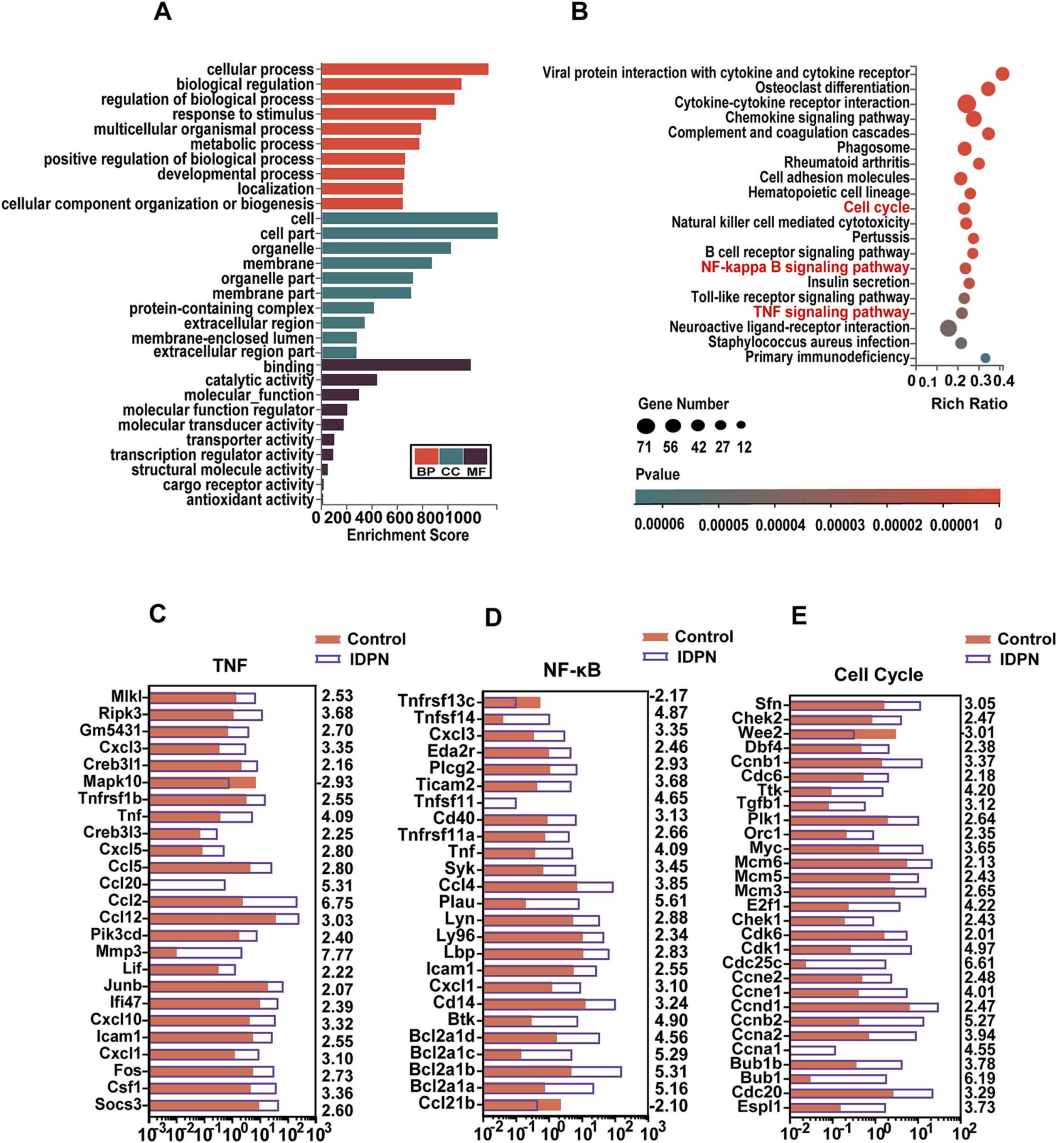
GO and KEGG analysis of DEGs in RNA-seq data. **(A)** GO analysis of the DEGs. BP, Biological process. CC, Cellular component. MF, Molecular function. **(B)** KEGG analysis of the DEGs in IDPN. **(C–E)** Expression levels of 25 DEGs in the TNF signaling pathway **(C)**, and 25 DEGs in the NF-ΚB signaling pathway **(D)**, and 29 DEGs in cell cycle **(E)**. The numbers on the right indicated log2 (fold change) of up-regulated and down-regulated gene expression in Control group compared with IDPN group.

To analyze the possible roles of DEGs and signaling pathways involved in IDPN damage, we applied Gene ontology (GO) and Kyoto Encyclopedia of Genes and Genomes (KEGG) pathway enrichment analysis of DEGs [log2(fold change) > 2, Q value<0.05]. By GO enrichment analysis, we found that the genes with altered expression in the IDPN group are highly enriched within functions including regulation of biological process, response to stimulus, protein-containing complex, catalytic activity and transcription regulator activity (Figure 5A). KEGG pathway enrichment analysis revealed that DEGs were highly enriched in immune-related signaling pathways, tumor necrosis factor (TNF) signaling pathways associated with inflammation and apoptosis, and nuclear factor *κ*-B (NF-κB) signaling pathways and in Cell Cycle (Figure 5B). As shown in Figures 5C, 24 genes and 1 gene in the TNF signaling pathway intercellular are significantly up-regulated and down-regulated in IDPN group, respectively. In the NF-κB signaling pathway, 23 genes and 2 genes were significantly up-regulated and down-regulated in IDPN group, respectively (Figure 5D). A total of 28 genes and 1 gene in cell cycle pathway were significantly upregulated and downregulated, respectively (Figure 5E).

**Figure 6:**
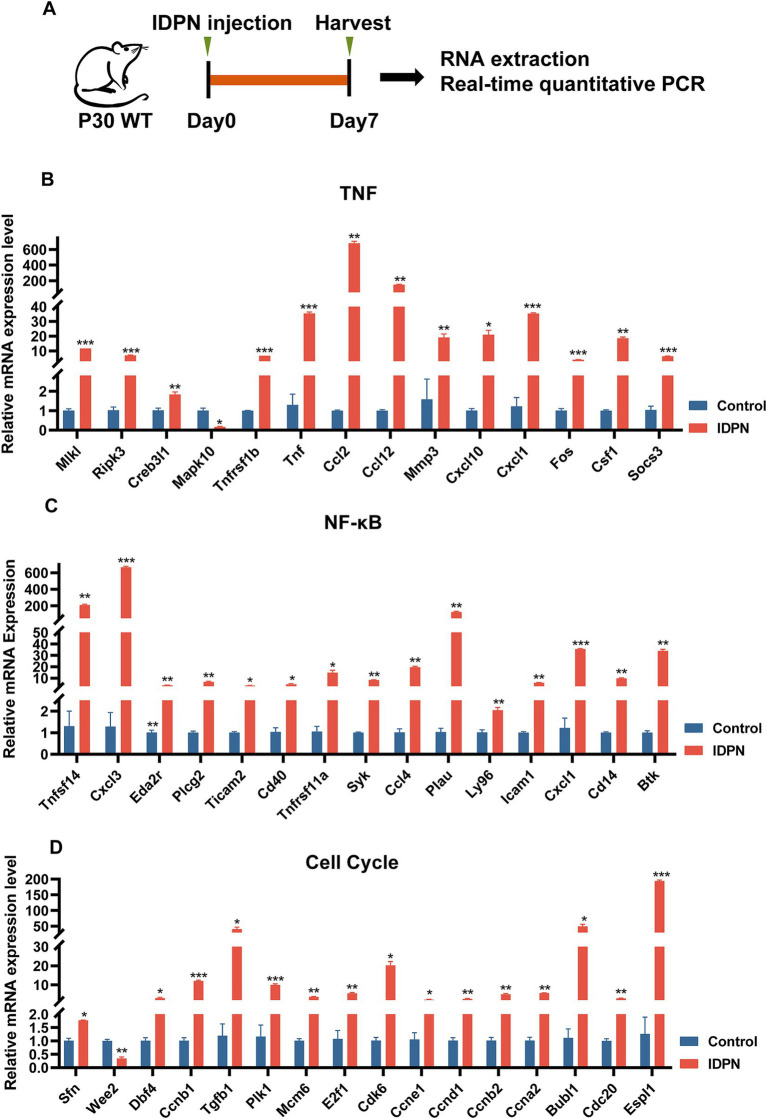
Quantification of the expression of signaling pathway genes. **(A)** The flow diagram for IDPN injection and utricle collection at P7 for Real-time QPCR. **(B–D)** Verification of expression level of DEGs in TNF signal pathway **(B)**, NF-κB signal pathway **(C)**, and cell cycle **(D)** by Real-time QPCR. *p < 0.05, **p < 0.01, ***p < 0.001. *n* = 3.

We then performed RT-QPCR (Figure 6A) to confirm the RNA-seq results, and found that the expression changes of 15 DEGs including Tnfsf14, Cxcl3, Eda2r, Plcg2, Ticam2, Cd40, Tnfrsf11a, Syk, Ccl4, Plau, Ly96, Icam1, Cxcl1, Cd14, and Btk in NF-κB pathway, 14 DEGs including Mlkl, RiPk3, Creb3l1, Tnfrsf1b, Tnf, Ccl2, Ccl12, Mmp3, Cxcl10, Cxcl1, Fos, Csf1, and Socs3 in TNF pathway, and 16 DEGs including Sfn, Wee2, Dbf4, Ccnb1, Tgfb1, Plk1, Mcm6, E2f1, Cdk6, Ccne1, Ccnd1, Ccnb2, Ccna2, Bubl1, Cdc20 and Espl1 in cell cycle, are consistent with RNA-seq data (Figures 6B–D). And most DEGs are upregulated. DEGs in NF-κB, TNF pathways and Cell Cycle suggests that these pathways may play an important role in IDPN-induced HCs damage. It is reported that inflammatory response is closely related to hearing loss caused by drugs, noise and age (Kalinec et al., 2017). Although NF-κB pathway, TNF pathway and cell cycle have not been fully studied in inner ear, they may be pathways that modulate HCs damage and need further study.

## Discussion

It has been reported that IDPN has neurotoxic and vestibulotoxic effect, and can be used to estabilish an *in vivo* damage model of vestibular dysfunction which shows a significant HCs loss phenotype (Schlecker et al., 2011). Here, we also found that IDPN injection *in vivo* showed a significant utricular HCs damage and vestibular dysfunction. Moreover, we found that IDPN injection caused much more utricular HCs loss and more pronounced vestibular behavioral disturbances than another commonly used neomycin damage model (Burns et al., 2012). However, the detailed mechanism behind IDPN damage is not well understood. Accordingly, we used RNA-seq to analyze the detailed gene expression profiles of utricles treated with IDPN. Among the highly expressed genes, Myelin protein zero (MPZ) is a member of the immunoglobulin gene superfamily with single extracellular, transmembrane and cytoplasmic domains(Shy et al., 2004). In a mouse model of noise-induced hearing loss, the Mpz gene was upregulated after noise exposure for 6 h (Maeda et al., 2015). Mpz gene were also up-regulated in our sequencing results. We further performed GO and KEGG analysis for DEGs to explore the pathways and genes that might play a role in the process. Recent studies have manifested that several DEGs regulate the cell death of utricular HCs. However, a large number of genes that may be potential targets for IDPN to damage vestibular hair cells have not been well studied which need further study in the future.

### DEGs in control and IDPN

We have shown that IDPN can effectively damage utricular HCs, and to determine the underlying mechanism behind this phenotype, we compared the gene expression levels between the IDPN group and the Control group. The roles of some DEGs have been previously reported. Among the upregulated DEGs in the IDPN group, some genes have been demonstrated to be associated with cell death or inner ear damage. Ccl2 plays a key role in ototoxic-induced sensorineural deafness and is involved in monocyte migration and activation after hearing injury (Chen et al., 2022; Sautter et al., 2006).

Other genes such as Ankrd1, Ccl6 and Fosl1 have been shown to promote cell death in other tissues. Ankrd1 acts as a co-activator of p53, modulating its transcriptional activity, and overexpression of ANKRD1 enhances cardiomyocyte apoptosis mediated by the up-regulation of p53 (Shen et al., 2015). CCL6 affects the pathogenesis of various inflammatory disorders by controlling the migration of macrophages and neutrophils (Coelho et al., 2007; Nakagawa et al., 2019). It is reported that high expression of CCL6 contributes to the H/R-induced apoptosis in H9c2 cells through enhancing the expression of IGF2-AS (Li et al., 2020). Fos-related antigen 1 (Fra-1) is encoded by the Fosl1 gene, and has been shown to be involved in various apoptosis processes (Xu et al., 2018a; Vaz et al., 2012; Bakiri et al., 2014).

Among the downregulated DEGs in the IDPN group, some had already been reported in the inner ear or were thought to be involved in cell death or inflammation. For example, overexpression of Olfm4 significantly inhibited the pro-inflammatory response of lung epithelial cells (Gong et al., 2021). N-deacetylase and N-sulfotransferase 4 (Ndst4) deficiency increased the basal level of apoptosis in the colonic epithelium (Jao et al., 2016). From otic placode induction to cellular differentiation in the organ of Corti, Fgf8 signaling plays roles in numerous steps of inner ear development (Ratzan et al., 2020). FGF8 signaling pathway can protect mesodermal cells from death, and loss of FGF8 can induce mesodermal cell death (Mariani et al., 2017; Montero et al., 2001; Diaz-Hernandez et al., 2021). Transmembrane channel like 2 (Tmc2) can maintain the normal sensory transduction of vestibular HCs, and sensory transduction is restricted to the extra-striolar region in absence of Tmc2 (Kawashima et al., 2011; Pan et al., 2013). In the cochlea, Tmc2 can partially compensate for Tmc1, but cannot replace it for a long time (Asai et al., 2018; Pan et al., 2013). Xin-actin binding repeat containing 2 (XIRP2) is essential for the long-term maintenance of HCs stereocilia. The deletion of Xirp2 in mice causes stereocilia degeneration and leads to hearing loss in mice (Scheffer et al., 2015; Francis et al., 2015).

Therefore, we found many DEGs have important roles in cell death and inflammation in HCs loss, which is consistent with our results and suggest that IDPN induced HCs damage may be involved with these HCs death and inflammation induced genes. However, there are much more DEGs that have not been reported and may play important roles in utricle HCs damage, which need further study in the future.

### Signaling pathway analysis

According to GO and KEGG enrichment analysis, we found that IDPN group contains more DEGs that play roles in regulation of biological process, response to stimulus, protein-containing complex, catalytic activity and transcription regulator activity. We studied the top 20 associated pathways in KEGG enrichment analysis, and IDPN injection may probably cause HCs damage through these enriched pathways, especially NF-κB signaling pathways and TNF pathways, which have been shown to be involved in inner ear inflammation and cell death processes and play an important role in inner ear dysfunction. It was reported that Siah3/NF-κB signaling pathway is activated in the cochlea and participates in the formation of cochlear inflammation after noise injury (Zhang et al., 2019a), And it has also been shown that expression of NF-κB increased in the utricle after cisplatin injury (Kim et al., 2008). Activation of TNF pathway is involved in the regulation cochlear HCs death and cochlear blood flow (Zhang et al., 2019b; Sharaf et al., 2016).

At the same time, it was reported that the synergistic effect of TNF pathway, NF-κB pathway and inflammatory signaling is associated with necroptosis (Chan et al., 2015; Thapa et al., 2013). Necroptosis can be activated by TNF death receptors, and appropriate NF-κB response promotes cell survival and reduce TNF cytotoxicity. At the same time, death receptors induced necrosis also can effectively induce pro-inflammatory transcription factor NF-κB (Papa et al., 2004; Kreuz et al., 2001). Recent studies have pointed out that pyroptosis may be activated by the levels of inflammatory factors such as TNF through the NF-κB-GSDMD axis (Jin et al., 2022). And it has been reported that there may be crosstalk between necroptosis and pyroptosis (Duong et al., 2015; Conos et al., 2017). Therefore, we speculate that IDPN injury may lead to a wider range of death pathways, such as necroptosis and pyroptosis, which may provide reference for scientific research using IDPN as a model for utricle damage.

A pro-inflammatory cytokine, TNF is involved in many biological processes, including necrosis, apoptosis, differentiation, and proliferation of cells (Liu, 2005; Pasparakis and Vandenabeele, 2015; Kalliolias and Ivashkiv, 2016; Huyghe et al., 2023). There are 14 DEGs verified in TNF pathway, and 13 DEGs are upregulated in IDPN group. These DEGs of the TNF pathway have been reported to be involved in the regulation of cell death and inflammation-related processes. Ripk3 with Mlkl is often used as a marker of necroptotic programmed cell death, and it has been suggested that RIPk3 and Mlkl can mediate necroptosis together (Zheng et al., 2014; Pasparakis and Vandenabeele, 2015). TNF is a central cytokine in the inflammatory response and induces inflammatory gene expression and cell death. Tnfrsf1b is the receptor of TNF, and controls local homeostatic effects (van Loo and Bertrand, 2023; Lightwood et al., 2021). MMP3 plays an important role in caspase-12 induced apoptosis, and its catalytic activity induces apoptosis (Muto et al., 2014). Fos is a member of the Fos family, and upregulation of Fos induces cell death (Bao et al., 2021; Xiao et al., 2018). Ccl12 is a small cytokine that attracts many types of immune cells. CCL12 overexpression enhances apoptosis, fibrosis and pyroptosis (Jia et al., 1996; Chen et al., 2023). Necroptosis can further amplify inflammation by increasing Ccl2 genetic expression, and inhibition of RIPK3 can reduce CCL2-mediated inflammation following intracebral hemorrhage (Huang et al., 2022; Xu et al., 2018b). CREB3L1 expression has been reported to be upregulated by endoplasmic reticulum stress, inhibiting cancer cell proliferation and promoting apoptosis (Yan et al., 2022). CSF1 can be a pro-inflammatory cytokine, and it is associated with a variety of inflammatory diseases (Saleh et al., 2018; Collison, 2018). SOCS3 is an established negative feedback regulation transcription factor, SOCS3 can promote adipocyte apoptosis by increasing inflammation and inhibiting the activity of JAK2/STAT3 signaling pathway (Liu et al., 2015).

Some of these DEGs also participate in the cell death process in the inner ear. Upregulation of Ripk3, Tnf, and MMP3 is involved in noise-induced hearing loss (Zheng et al., 2014; Fuentes-Santamaría et al., 2017; Park et al., 2014). Some studies found that the hearing threshold increased for a short time after noise and then decreased, during which Fos expression increased, which was considered to be related to the protection and survival of Corti (Shizuki et al., 2002). Some researchers found that CXCL10 may directly promote immune-mediated apoptosis in the inner ear, leading to age-related deafness. The expression of CXCL10 is increased in acute noise induced hearing loss, which promotes immune-mediated apoptosis in the ear and induces age-related deafness in humans (Dong et al., 2014; Oh et al., 2019). Dexamethasone systematically down-regulates the higher expression of Ccl12 in the noise-exposed cochlea, which may provide a basis for drug therapy for acute sensorineural hearing loss (Maeda et al., 2018).

It is well known that the death receptor in the TNF superfamily is one of the typical signals that activate necroptosis (Thapa et al., 2013a). However, cell death is not the only signaling outcome for the death receptors, and the signaling stimulation of the death receptor is often accompanied by NF-κB activation (Chan et al., 2015a). NF-κB usually induces the expression of pro-survival genes, which is mutually exclusive with apoptosis or necroptosis. Moreover, under certain conditions, TNF signaling can act in conjunction with NF-κB signaling to induce apoptosis or necroptosis and inflammation (Chan and Lenardo, 2000; Varfolomeev et al., 2007; Petersen et al., 2007).

The NF-κB signaling pathway is a highly conserved evolutionary pathway. This signaling pathway plays a key role in regulating immune and inflammatory responses, and it broadly influences cell survival, apoptosis, differentiation, and proliferation (Xiao and Ghosh, 2005; Hayden and Ghosh, 2008; Perkins, 1997). It was demonstrated that NF-κB is a key bridge for the expression of inflammatory cytokines and other mediators mediating inflammatory responses in many of the inflammatory and immune damage associated with the inner ear (Fetoni et al., 2019; Sai et al., 2020; Ferreiro and Komives, 2010).

In our data, 15 DEGs in the NF-κB pathway was verified. These DEGs have been shown to be involved in inflammation and cell death in the inner ear. As a co-receptor of Toll-like receptors, CD14 can activate NF-κB and a series of pro-inflammatory cytokines, such as TNF-*α*, IL-1*β*, thereby causing changes in the microenvironment (Li et al., 2013; Fu et al., 2021). CD14-positive inflammatory cells into the cochlea were briefly increased in capsaicin-induced transient hearing loss (Mukherjea et al., 2011). CD40, a member of the tumor necrosis factor receptor (TNFR) family, can activate NF-κB and play an important role in cell survival, cell death, immune response, and inflammatory diseases (Jundi et al., 2012; Elgueta et al., 2009). Pro-inflammatory chemokine Ccl4, which belongs to CC chemokine family, is regulated by NF-κB and plays an indispensable role in various inflammatory diseases and tissue damage (Ahmad et al., 2019; Son et al., 2007). Ccl4 expression is up-regulated in cochlear inflammatory damage induced by noise exposure (Tornabene et al., 2006). ICAM-1 is part of the immunoglobulin superfamily, and it is also associated with a variety of inflammatory diseases and conditions (Springer, 1990; Roebuck and Finnegan, 1999). NF-κB signals can induce ICAM-1, which plays a key role in innate and adaptive immune responses (Ghosh and Karin, 2002). ICAM1 acts as an adhesion molecule that interacts with receptors on the surface of immune cells, allowing it to exfiltrate into the cochlea. Upregulated expression of ICAM-1 was observed in both noise exposure and lipopolysaccharide-induced inner ear inflammation, leading to further hearing impairment (Tornabene et al., 2006; Bae et al., 2021). Cxcl1 expression is up-regulated to participate in the inflammatory response (Korbecki et al., 2022). In the inner ear, CXCL1 was found to be expressed in mouse vestibular cells (Elkon et al., 2015). In hearing loss induced by noise exposure, the expression level of proinflammatory cytokines CXCL1 in the inner ear of mice was significantly increased (Landegger et al., 2019; Maeda et al., 2018). The other genes have been shown to have pro-inflammatory or mediating cell death roles in other tissue cells. EDA2R could upregulate the NF-κB family members including RELA (Verhelst et al., 2015). TNFRSF11A, also known as receptor activator of NF-κB (RANK), belongs to the TNFR family and has an extracellular domain highly similar to CD40. It can activate various signaling pathways such as NF-κB, JNK, ERK, p38α, and Akt/PKB (von dem Knesebeck et al., 2012; Darnay et al., 1998). TNFSF14 is a member of the TNF family, also known as lymphotoxin-related inducible ligand (LIGHT) or CD258, which can induce both typical and atypical pathways of NF-κB non-canonical and canonical pathway (Qiu et al., 2014; Mauri et al., 1998; Jin et al., 2010). A recent study showed that TNFSF14 in combination with IFN-*γ* induces apoptosis of islet β cells via an intrinsic mitochondrial pathway through NF-κB-mediated regulation of anti-apoptotic or pro-apoptotic Bcl-2 family member expression (Zheng et al., 2016). Activation of SYK triggers a variety of signaling pathways, including the NF-κB signaling pathway, and there is growing evidence that SYK is involved in inducing cell death and some inflammatory damage (Xu et al., 2019; Yin et al., 2016; Chen et al., 2021a; Lee et al., 2016; Bukong et al., 2016; LaRocca et al., 2015). High expression of PLCG2 has also been shown to contribute to pro-inflammatory signaling (Zhang et al., 2023; Tsai et al., 2022). The TIR domain of TICAM-2 is recruited by Toll-like receptor 4 to initiate the activation of NF-κB, which then promotes the release of downstream proinflammatory cytokines in inflammatory microglia (Zhao et al., 2020). BTK belongs to the TEC tyrosine kinase family, and up-regulation of BTK expression can activate the NF-κB pathway (Li et al., 2023). It has been shown that treatment with the BTK inhibitor ibrutinib attenuates the activation of NF-κB and NLRP3 inflammasome (Purvis et al., 2020). CXCL3 can be activated by activation of the NF-κB signaling cascade to further promote the expression of pro-inflammatory factors (Reyes et al., 2021; Gulati et al., 2018; Zhou et al., 2022). LY96 can activate macrophage-mediated NF-κB and STAT3 pathways and also promote the release of pro-inflammatory cytokines and adhesive molecules. High expression of LY96 has been proven to be associated with a sustained pro-inflammatory immune response (Jin et al., 2020; Rajamanickam et al., 2020; Jiang et al., 2023).

In our sequencing results, we found that DEGs were also highly enriched in the cell cycle pathway. 16 genes in cell cycle are verified by QPCR to be differentially expressed in IDPN group, and most of them are upregulated. Among them, upregulation of Sfn, Dbf4, Ccnb1, Tgfb1, Plk1, Mcm6, E2f1, Cdk6, Ccne1, Ccnd1, Ccnb2, Ccna2, Bubl1, Cdc20 and Espl1are reported to induce cell cycle (Bruno et al., 2022; Tachibana et al., 2005; Stoeber et al., 2001; Umate et al., 2011; Rizou et al., 2018; Kaplan et al., 2017; Sun et al., 2009; Nasmyth and Haering, 2009; Uhlmann, 2001; Suski et al., 2021). It seems that the cell cycle was upregulated overall, which is not consistent with the HCs death phenotype we observed. However, it is reported by several articles that damage of HCs could induce the spontaneous HC regeneration to some extent (Cox et al., 2014; Bramhall et al., 2014; White et al., 2006; Oesterle et al., 2008; Forge et al., 1993; Warchol et al., 1993; Zhang et al., 2017a), and in the utricle treated by IDPN, there is not only the loss of HCs, but also the proliferation and differentiation of supporting cells (Zeng et al., 2020), which may explain the overall upregulation of cell cycle observed in this study. However, we do not know much about the operation of the specific mechanisms of this proliferative capacity. On the other hand, cell cycle reentry of mitotic cells is an early manifestation of apoptosis (Freeman et al., 1994; Herrup and Busser, 1995; Padmanabhan et al., 1999), and thus cell cycle-related genes may also participate in the death process of HCs.

## Conclusion

In summary, we demonstrated that IDPN injection *in vivo* resulted in a more pronounced HCs injury in the utricle and more severe vestibular dysfunction than neomycin injection *in vivo*. We characterized the transcriptome changes in IDPN-induced utricle injury model, and identified key genes and pathways involved in this process. These pathways and genes may be the key regulators of HCs injury and vestibular dysfunction, which may be the target genes for further study of mechanisms of HCs protection.

## Data Availability

The data presented in the study are deposited in the SRA repository, accession number PRJNA1193838.

## Footnotes

^1^ http://www.geneontology.org/

^2^ https://www.kegg.jp/

^3^ https://en.wikipedia.org/wiki/Hypergeometric_distribution

^4^ https://biosys.bgi.com
